# Negative prognostic impact of regulatory T cell infiltration in surgically resected esophageal cancer post-radiochemotherapy

**DOI:** 10.18632/oncotarget.4428

**Published:** 2015-06-10

**Authors:** Erika Vacchelli, Michaela Semeraro, David P. Enot, Kariman Chaba, Vichnou Poirier Colame, Peggy Dartigues, Aurelie Perier, Irene Villa, Sylvie Rusakiewicz, Caroline Gronnier, Diane Goéré, Christophe Mariette, Laurence Zitvogel, Guido Kroemer

**Affiliations:** ^1^ Gustave Roussy Cancer Campus, Villejuif, France; ^2^ INSERM, U1138, Paris, France; ^3^ Equipe 11 Labellisée par la Ligue Nationale contre le Cancer, Centre de Recherche des Cordeliers, Paris, France; ^4^ Université Paris Descartes/Paris V,Sorbonne Paris Cité, Paris, France; ^5^ Université Pierre et Marie Curie/Paris VI, Paris, France; ^6^ INSERM, U1015, Villejuif, France; ^7^ Center of Clinical Investigations in Biotherapies of Cancer (CICBT) 1428, Villejuif, France; ^8^ Metabolomics and Cell Biology Platforms, Gustave Roussy Cancer Campus, Villejuif, France; ^9^ Department of Pathology, Gustave Roussy Cancer Campus, Villejuif, France; ^10^ Digital Pathology, Departement of Pathology, Gustave Roussy Cancer Campus, Villejuif, France; ^11^ Department of Digestive and Oncological Surgery, Claude Huriez University Hospital, Lille, France; ^12^ North of France University, Lille, France; ^13^ Department of Surgical Oncology, Gustave Roussy Cancer Campus, Villejuif, France; ^14^ Faculté de Médecine, Université Paris-Sud/Paris XI: Kremlin-Bicêtre, France; ^15^ Pôle de Biologie, Hôpital Européen Georges Pompidou, AP-HP, Paris, France

**Keywords:** immunogenic cell death, autophagy, ATG16L1, pattern recognition receptor, apoptosis

## Abstract

Ever accumulating evidence indicates that the long-term effects of radiotherapy and chemotherapy largely depend on the induction (or restoration) of an anticancer immune response. Here, we investigated this paradigm in the context of esophageal carcinomas treated by neo-adjuvant radiochemotherapy, in a cohort encompassing 196 patients. We found that the density of the FOXP3^+^ regulatory T cell (Treg) infiltrate present in the residual tumor (or its scar) correlated with the pathological response (the less Tregs the more pronounced was the histological response) and predicted cancer-specific survival. In contrast, there was no significant clinical impact of the frequency of CD8^+^ cytotoxic T cells. At difference with breast or colorectal cancer, a loss-of-function allele of toll like receptor 4 (*TLR4*) improved cancer-specific survival of patients with esophageal cancer. While a loss-of-function allele of purinergic receptor P2X, ligand-gated ion channel, 7 (*P2RX7*) failed to affect cancer-specific survival, its presence did correlate with an increase in Treg infiltration. Altogether, these results corroborate the notion that the immunosurveillance seals the fate of patients with esophageal carcinomas treated with conventional radiochemotherapy.

## INTRODUCTION

There is growing awareness of the fact that cancer is not just a cell-autonomous disease determined by accumulating (epi)genetic aberrations in transforming and neoplastic cells. Rather, cancer is also a systemic disease in thus far that it only occurs upon failure of the immunosurveillance system [1, 2]. Moreover, in spite of the fact that cytotoxic or so-called targeted therapies have been conceived as having purely cell-autonomous effects, it has become increasingly clear that they only can extend patient survival on the long-term if they succeed in reinstating immunosurveillance [3-5].

The importance of immunosurveillance has been particularly well documented for breast cancer, perhaps driven by its high frequency, as well as the fact that neo-adjuvant and adjuvant chemotherapies do have long-term effects and significantly extend overall survival of patients with this pathology [6, 7]. The efficacy of neo-adjuvant chemotherapy of mammary carcinomas is increased by pre-existing anticancer immune response, as indicated by the presence of CD8^+^ cytotoxic T lymphocytes (CTL) in the tumor bed at diagnosis [6-9]. Moreover, an amelioration of the ratio between CD8^+^ CTL and FOXP3^+^ regulatory T cells (Tregs) after one cycle of anthracycline-based chemotherapy is able to predict the pathological complete response after six cycles of chemotherapy [10, 11].

Additional immunological relevant factors that affect the prognosis of breast cancer patients include loss-of-function alleles of pattern recognition receptors (such as toll like receptor 4 (*TLR4)* and purinergic receptor P2X, ligand-gated ion channel, 7 (*P2RX7*) [12, 13] and the local expression of genes [such as myxovirus (influenza virus) resistance 1 (*MX1*)]) that are tied to the type 1 interferon response [14]. While loss-of-function mutations affecting *TLR4* or *P2RX7* have a negative impact on the survival of breast cancer patients [12, 13], local expression of *MX1* constitutes a positive prognostic marker [14]. Nonetheless, these biomarkers do not have a universal impact on cancer patient survival. For example, we found that loss-of-function alleles of *TLR4* and *P2RX7* have no significant impact on the survival of patients with non-small cell lung cancer [15].

Driven by these considerations, we decided to investigate the influence of immunological parameters on the therapeutic response and survival of patients with esophageal carcinoma. We demonstrate that the frequency of Tregs determined in the surgical specimen after radiochemotherapy has a major impact on the pathological and clinical response.

## RESULTS AND DISCUSSION

### Paradoxical effect of the loss-of-function *TLR4* allele Asp299Gly on esophageal cancer survival

**Figure 1:**
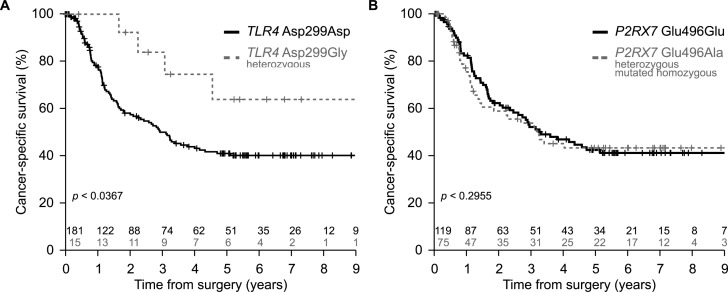
Impact of *TLR4* and *P2RX7* loss-of-function alleles on cancer-specific survival in esophageal cancer **A.** Kaplan-Meier of the cancer-specific survival estimated in a cohort of esophageal cancer patients (*n* = 196) treated with neo-adjuvant cisplatin-based radiochemotherapy and bearing *TLR4* rs4986780 with AA (wild type, Asp299) or AG (heterozygous, Asp299Gly) genotype. **B.** Kaplan-Meier of the cancer-specific survival estimated in a cohort of esophageal cancer patients (*n* = 194) treated with neo-adjuvant radiochemotherapy and bearing *P2RX7* rs3751143 with AA (wild type, Glu496) or AC (heterozygous, Glu496Ala) + CC (mutated homozygous, Ala496Ala) genotypes. Statistical significance was determined by likelihood ratio test (LRT).

As mentioned in the introduction, loss-of-function alleles of *TLR4* (rs4986790, Asp299Gly) and *P2RX7* (rs3751143, Glu496Ala) may negatively affect the therapeutic response of breast cancer patients to adjuvant chemotherapy [12, 13]. Moreover, the most common loss-of-function allele of *TLR4* (Asp299Gly) also is a poor prognostic feature for colorectal cancer patients treated with adjuvant chemotherapy [16]. Given these premises, we determined the impact of such loss-of-function alleles on the survival of 196 esophageal cancer patients that were treated with neo-adjuvant radiochemotherapy (Table 1, Supplemental Figure 1). To our surprise, we found that patients harboring one copy of the mutated allele of *TLR4* exhibited an improved cancer-specific survival as compared to the majority of patients bearing two copies of the most frequent, functional *TLR4* allele (Figure 1A). In contrast, loss-of-function alleles affecting *P2RX7* (Glu496Ala, Figure 1B), the autophagy-relevant gene autophagy related 16-like 1 (*S. cerevisiae*) (*ATG16L1,* rs2241880, Thr300Ala) (Supplemental Figure 2A) and the autoimmune disease-related gene protein tyrosine phosphatase, non-receptor type 22 (lymphoid) (*PTPN22,* rs2476601, Arg620Trp) (Supplemental Figure 2B) had no impact on patients survival. These findings are reminiscent of those previously obtained on patients with non-small cell lung cancer, in which loss-of-function mutation of *TLR4* had positive effects while that affecting *P2RX7* had no impact [15].

**Table 1:** Clinical and histopathology characteristics of the 196 esophageal cancer patients

Cohort parameters	Variable	n (%)
Clinical parameters		
Gender		
	M	177 (90.3)
	F	19 (9.7)
Smokers		
	No	19 (9.7)
	Yes	167 (85.2)
	Missing	10 (5.1)
Alcoholics		
	No	21 (10.7)
	Yes	125 (63.8)
	Missing	50 (25.5)
Denutrition		
	No	136 (69.4)
	Yes	60 (30.6)
Tumor related parameters		
T of TNM		
	T0	64 (32.7)
	T1	22 (11.2)
	T2	23 (11.7)
	T3	63 (32.1)
	T4	18 (9.2)
	Missing	6 (3.1)
N of TNM		
	N0	116 (59.2)
	N1	54 (27.6)
	N2	20 (10.2)
	N3	6 (3.1)
M of TNM		
	M0	186 (94.9)
	M1	9 (4.6)
	Missing	1 (0.5)
Metastasis localization		
	No	186 (94.9)
	Liver	3 (1.6)
	Lung	3 (1.5)
	Stomac	3 (1.5)
	Missing	1 (0.5)
Site		
	Neck	5 (2.6)
	Cervical	10 (5.1)
	Thoracical	38 (19.4)
	Mid Third	105 (53.6)
	Lower Third	38 (19.4)
Histological differentiation		
	High	108 (55.1)
	Mid	36 (18.4)
	Low	11 (5.6)
	Missing	41 (20.9)
Response to treatment		
	Complete response	55 (28.1)
	Partial response	102 (52.0)
	Stable disease	30 (15.3)
	Progressive disease	6 (3.1)
	Missing	3 (1.5)
Mandard classification (TRG)		
	Grade 1	64 (33)
	Grade 2	23 (12)
	Grade 3	33 (17)
	Grade 4	56 (28)
	Grade 5	20 (10)

Abbreviations: F, female; M, male; n, number; TNM, Tumor-Node-Mestastais; TRG, tumor regression grade.

### Absent (or paradoxical) effects of CD8^+^ T lymphocytes infiltration on patients survival

**Figure 2:**
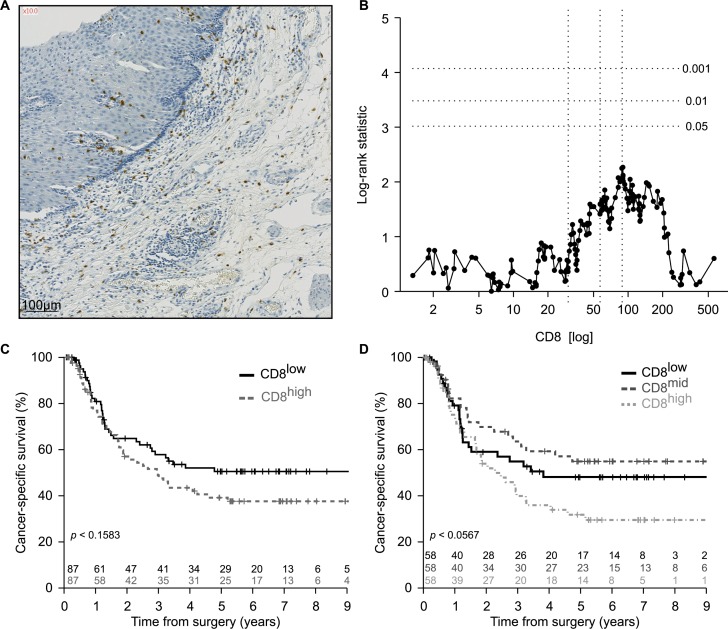
Enumeration of CD8^+^ infiltrating lymphocytes in esophageal cancer **A.** Representative picture of immunohistochemical staining of primary paraffin embedded esophageal carcinoma using CD8 specific antibody. Positive cells are stained brown. The exact number of CD8^+^ lymphocytes was evaluated in the tumor site and in the surrounding healthy or cicatricial tissue. **B.** Plot of the log-rank statistics at all possible values of CD8^+^ lymphocytes infiltration used to establish a cut-off that optimally separate the cohort into 2 prognostic groups. *p* values are simulated by Monte Carlo sampling (B=1999) and approximated log-rank statistics corresponding to the *p* < 0.05, *p* < 0.01 and *p* < 0.001 significance thresholds drawn as dotted horizontal lines. **C.**, **D.** Kaplan-Meier of the cancer-specific survival (*n* = 174) according to the median **C.** or terciles **D.** of CD8^+^ infiltrating lymphocytes at the time of surgery.

There is an abundant – and partially contradictory – literature on the role of CTL in esophageal cancer. Thus, the presence of CD8^+^ T lymphocytes within tumor nodules before treatment has been interpreted as having a positive impact on overall survival [17-21]. In contrast, according to one study, macrophage infiltration was positively associated with CD8^+^ T lymphocyte infiltration, yet negative associated with cancer-specific survival in esophageal adenocarcinoma [22]. Here, we investigated the rather heterogeneous density of the CD8^+^ T cell infiltrate within the residual tumor (or in the case of its complete disappearance within the cicatricial tissue) post-radiochemotherapy (Figure 2A). Separation of the cohort according to the median (or any other threshold) yielded no significant differences between CD8^low^ and CD8^high^ tumors with regard to patient survival (Figure 2B, 2C). However, separation of the cohort into three terciles results in a statistical trend (*p* < 0.0567) suggesting that high infiltration by CD8^+^ T cells might have a negative impact on patient survival (Figure 2D). At this stage, we ignore whether this absent (or paradoxical) effect of the CD8^+^ T cell infiltrate on patients survival reflects the presence of dysfunctional (anergy or exhausted) T cells in the tumor bed post-radiochemotherapy.

### Negative impact of FOXP3^+^ T cells on the survival of esophageal cancer patients

**Figure 3:**
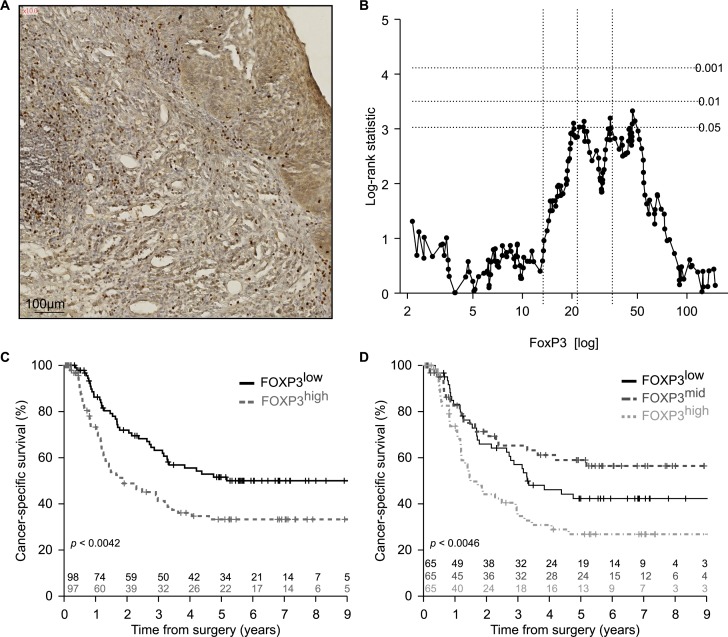
Enumeration of FOXP3^+^ T regulatory infiltrating lymphocytes in esophageal cancer **A.** Representative picture of immunohistochemical staining of primary paraffin embedded esophageal carcinoma using FOXP3 specific antibody. Positive cells are stained brown. The exact number of FOXP3^+^ lymphocytes was evaluated in the tumor site and in the surrounding healthy or cicatricial tissue. **B.** Plot of the log-rank statistics at all possible values of FOXP3^+^ lymphocytes infiltration used to establish a cut-off that optimally separate the cohort into 2 prognostic groups. *p* values are simulated by Monte Carlo sampling (B=1999) and approximated log-rank statistics corresponding to the *p* < 0.05, *p* < 0.01 and *p* < 0.001 significance thresholds drawn as dotted horizontal lines. **C**.,**D**. Kaplan-Meier of the cancer specific-survival (*n* = 195) according to the median **C.** or terciles **D.** of FOXP3^+^ infiltrating lymphocytes at the time of surgery.

**Figure 4:**
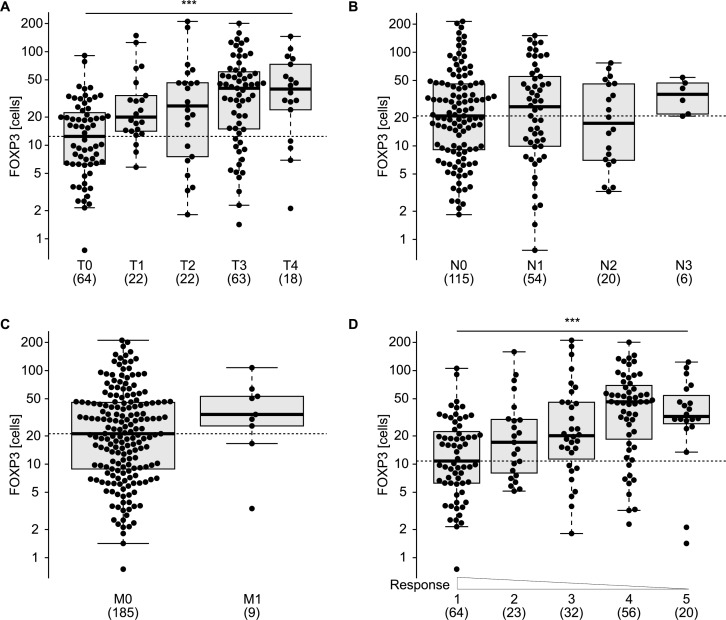
Distribution of FOXP3^+^ T regulatory infiltrating lymphocytes according to clinical parameters and treatment response Enumeration of FOXP3^+^ cells according to tumor staging at the time of surgery **A.**, lymph nodes involvement **B.**, presence of metastasis **C.**, tumor regression grading (TRG) **D.**. *** *p* < 0.001 (one way ANOVA test).

**Figure 5:**
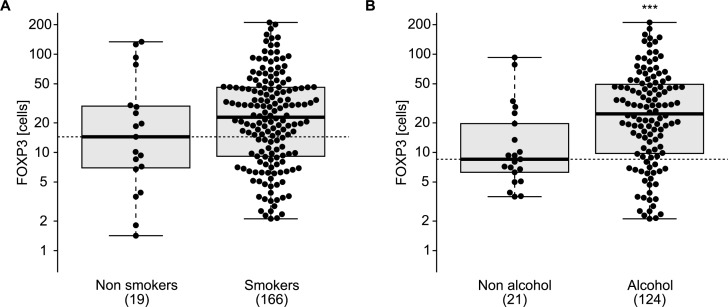
Correlation of FOXP3^+^ T regulatory infiltrating lymphocytes and comorbidities FOXP3^+^ T regulatory lymphocytes infiltrating tumor or cicatritial tissue were associated with smoking status **A.** or alcohol consumption **B.** in esophageal cancer patients. ns, non significant, *** *p* < 0.001 (unpaired Student's t test).

**Figure 6:**
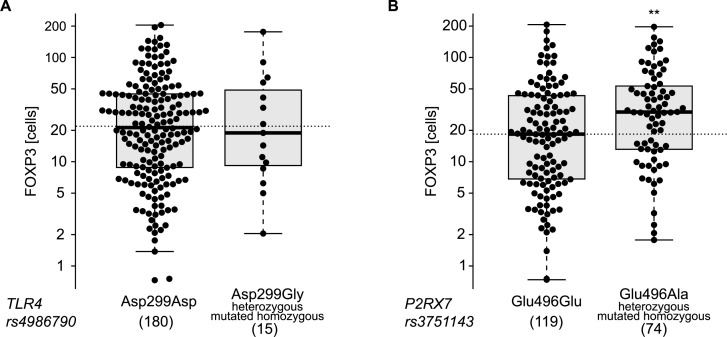
Single nucleotide polymorphisms (SNPs) association with infiltrating FOXP3^+^ lymphocytes Distribution of FOXP3^+^ lymphocytes levels across the genoptypes of *TLR4* (rs4986790) **A.** and *P2RX7* (rs3751143) **B.**. Each dot represents one patient. ** *p* < 0.01 (unpaired *t*-test).

There are numerous reports describing an increment in the frequency of Tregs among tumor-infiltrating lymphocytes in esophageal cancers [23-25] and that this increase may have a negative prognostic impact [26]. This phenomenon augments with tumor stage [27, 28] and has a negative impact on overall survival [26]. Radiochemotherapy reduces the frequency of Tregs in the center of tumor lesions [29], and this decrease correlates with the median survival of the patients [30], perhaps because of an improvement of immunosurveillance tied to Treg depletion. In accord with the published literature, we observed heterogeneity of the FOXP3^+^ Treg infiltrate post-radiochemotherapy (Figure 3A), as well as a negative correlation between the density of the Treg infiltrate and cancer-specific survival (Figure 3B-3D). The frequency of FOXP3^+^ T cells infiltrating the tumor (or its scar) post-radiochemotherapy increased with tumor staging (Figure 4A) but was not influenced by the number of lymph node or distant metastases (Figure 4B, 4C). The degree of the histological response to radiochemotherapy, as assessed according to tumor regression grade (TRG) system [31] (that attributes the lowest number to the best response), also exhibited a negative correlation with the Treg infiltrate, meaning that tumors showing a complete regression (TRG 1) contained less Tregs than tumors presenting incomplete (TRG 2-4) or absent (TRG 5) responses (Figure 4D). In sharp contrast, the CD8^+^ infiltrate did not correlate with any of these clinical parameters (Supplemental Figure 3). Smokers and non-smokers did not differ in the density of the FOXP3^+^ infiltrate (Figure 5A), while alcoholism apparently increased this parameter (Figure 5B). While there was no difference in the frequency of tumor-infiltrating Tregs among patients with normal or loss-of-function alleles of *TLR4* (Figure 6A), patients bearing the mutated alleles of *P2RX7* exhibited an increased Treg infiltration (Figure 6B). Although this latter parameter does not improve risk stratification of esophageal carcinoma patients (Figure 1B), it may indicate some impact of purinergic receptor signaling on Tregs function [32].

### Concluding remarks

The present paper adds to a series of reports on the relevance of immunosurveillance for the fate of patients afflicted by esophageal carcinoma. The preponderant vision of the field is that this kind of malignancy is subjected to immunosurveillance by CTL. Indeed, there is some evidence for CTL-mediated immunoediting, meaning that esophageal squamous cell carcinomas tend to lose proteins required for the expression of MHC class I molecules (such as Δ-2 microglobulin, tapasin-1 and HLA class I) [33]. However, this phenomenon, which correlated with a reduction in CTL infiltration, had no impact on the clinical course of the disease [34]. Additionally, neo-adjuvant chemotherapy of esophageal squamous cell carcinoma with 5-fluorouracil and cisplatin increases HLA class I expression as it induces infiltration by CD8^+^ T lymphocytes in the stroma [35].

When characterizing the immune infiltrate of esophageal carcinomas post-radiochemotherapy, we observed that the number of CD8^+^ T cells found in the residual tumor or the scar was irrelevant for patient survival. In sharp contrast, it appeared that the frequency of FOXP3^+^ Tregs consistently correlated with the therapeutic response (meaning that few FOXP3^+^ T cells were found in those residual lesions that had responded to radiochemotherapy) as well as with the cancer-specific survival.

Previous work revealed that the frequency of Tregs is positively correlated with their apoptosis resistance [36], suggesting that their frequency is dictated by Treg-intrinsic parameters. However, it has also been shown that the number of Tregs is positively influenced by the presence of the chemokines (C-C motif) ligand (CCL) 17 and CCL22 in the tumor microenvironment [37], indicating that extrinsic factors may control their recruitment, differentiation or survival within the neoplastic lesion. We found that the level of expression of nuclear high mobility group box 1 (HMGB1) and cytoplasmic microtubule-associated protein 1 light chain 3 beta (MAP1LC3B best known as LC3B) *puncta* had no impact on the survival of patients with esophageal cancer (Supplemental Figure 4), nor on the frequency of intratumoral Tregs (not shown), suggesting that these factors (which may affect Treg infiltration in other tumors) [38, 39] have no impact on Treg biology in the context of esophageal carcinoma. As a caveat, however, it has to be noticed that the examination of such markers of cellular stress and local immune function has only been performed at one time point, on surgical specimen post-radiochemotherapy. Hence, this delayed ‘snapshot’ might hide the entire, likely complex sequence of tissue reactions that occur between the radiochemotherapeutic intervention and the surgical removal of the tumor.

Irrespective of the multiple incognita that the present study fails to illuminate, there is convincing evidence that Treg infiltration post-radiochemotherapy may be assessed as an independent prognostic factor for an ever more accurate risk stratification of esophageal carcinomas. This result must be confirmed in an independent, prospective multicenter study before its diagnostic implementation.

## MATERIALS AND METHODS

### Patients and study design

One-hundred-night-six esophageal cancer patients were enrolled into the study at the Claude Huriez University Hospital (Lille, France) and written informed consent was obtained from all the included patients according to the local ethical committee. All patients received neo-adjuvant radiochemotherapy consisting of 3 cycles of 5-fluorouracil plus cisplatin or oxaliplatin combined with 45 Gys (median dose) of radiotherapy. Patient characteristics are depicted in Table 1. Finally, not all the patients were exploitable for each statistical analysis due to the inadequate amount and quality of samples or due to missing information in the clinical database.

### Genotyping

Genomic DNA was isolated from formalin fixed paraffin–embedded (FFPE) esophageal cancer tissue blocks by means of the DNeasy Blood and Tissue Kit (Qiagen, Valencia, CA, USA) and stored at −20°C in Tris-EDTA (TE) buffer. Gene-specific Taqman^®^ primers and genotype-specific probes (Applied Biosystems-Life Technologies Inc.) were used to amplify (i) a *TLR4* fragment containing the Asp299Gly single nucleotide polymorphism (SNP) (rs4986790), (ii) a *P2RX7* fragment containing the Glu496Ala SNP (rs3751143), (iii) an *ATG16L1* fragment containing the Thr300Ala SNP (rs2241880) and a *PTPN22* fragment containing the Arg620Trp SNP (rs2476601). All selected polymorphisms were amplified in a multiplex PCR performed on StepOnePlus Real-Time (RT) PCR System (Applied Biosystems-Life Technologies Inc.). Genotypes were determined by comparing the signals from fluorescent probes (FAM and VIC) and by calculating the natural logarithm of the ratio between the FAM and VIC signals [log (FAM/VIC)].

### CD8 and FOXP3 immunohistochemical staining

Paraffin–embedded esophageal cancer specimens were assessed for the infiltration by CD8^+^ (Novocastra, Newcastle, UK, M-NCL CD8295) performed on a BenchMark XT automated immunostainer (Ventana, Tucson, AZ, USA). Antigen retrieval was performed by incubating slides in EDTA buffer (pH 8.0) for 30 min at 95°C, then the primary antibody was incubated for 30 min. FOXP3^+^ regulatory T cells staining was manually assessed using the Novolink Kit (Menarini Diagnostics, RE7140-K). FFPE cancer tissue sections were deparaffinized for antigen retrieval and then rehydrated through graded alcohols to water. Antigen retrieval was carried out by heating slides for 30 min in pH 9.0 citrate buffer at 98 °C. The sections were incubated for 5 min with the Peroxidase Block reagent, and subsequently washed twice for 5 min with PBS. Following incubation for 5 min at room temperature with the Protein Block reagent, tissue sections were washed twice for 5 min with PBS, and then incubated 1 hour at room temperature with a primary antibody specific for FOXP3 regulatory T cell (ab450, Abcam) at the final concentration of 4μg/mL. After two washes with PBS, sections were incubated for 30 min with the Post Primary Block reagent, washed again as before and incubated for 30 min with Polymer secondary antibodies. Upon two additional washes, secondary antibodies were revealed with the liquid DAB Substrate Chromogen system (10 min incubation). Finally, slides were washed in distilled water, and counterstained with hematoxylin.

### Immunohistochemical detection of HMGB1

FFPE cancer tissue sections were deparaffinized in changes of xylène and rehydrated in absolute ethanol. Antigen retrieval was carried out by heating slides for 30 min in pH 6.0 citrate buffer at 98 °C. Endogenous peroxidase activity was inhibited by incubation with 3% hydrogen peroxidase (Dako, Trappes, France) for 15 min and then washed twice for 5 min with 0.025% Triton (v/v in TBS). Sections were then saturated 20min with Protein Block Serum Free (Dako, Trappes, France). Without washing, the primary antibody, a polyclonal rabbit anti HMGB1 antibody (ThermoScientist Pierce, PA1-16926) at the final concentration of 4μg/mL, was incubated overnight. After two washes in 0.025% Triton (v/v in TBS) sections were incubated by the secondary antibody (En vision-Rabbit, Dako, Trappes, France) for 45min, Upon two additional washes the peroxidase activity was revealed by means of daminobenzidine (DAB) substrate (Dako, Trappes, France), and the sections were counterstained with Mayer's hematoxylin.

### Immunohistochemical detection of LC3B

Immunohistochemical staining of cancer tissue sections was performed using the Novolink Kit (Menarini Diagnostics, RE7140-K). Tissue slides were deparaffinized in changes of xylène and rehydrated in decreasing concentrations of absolute ethanol (100%, 95%, 80%, 70% and 50%). Antigen retrieval was carried out by heating slides for 30min in pH 6.0 citrate buffer at 95°C. Thereafter, sections were incubated for 5min with the Peroxidase block reagent, and subsequently washed twice for 5min with 0.1% Tween 20 (v/v in PBS). Following a 5-min-long incubation at room temperature with the Protein block reagent, tissue sections were washed twice for 5min with 0.1% Tween 20 (v/v in PBS), and then incubated overnight at 4°C with a primary antibody specific for LC3B (clone 5F10, Nanotools, 0231-100), dissolved in 1% bovine serum albumin (w/v in TBS) at the final concentration of 25 μg/mL. This antibody recognizes both the soluble (LC3-I) and the membrane-bound form (LC3-II) of LC3B. After two washes in 0.1% Tween 20 (v/v in PBS), sections were incubated for 30min with the Post Primary Block reagent, washed again (as previously described) and incubated for 30 min with the horseradish peroxidase-coupled polymer secondary antibodies. Upon two additional washes, secondary antibodies were revealed with the liquid DAB Substrate Chromogen system (10min incubation). Finally, slides were washed in distilled water, and counterstained with hematoxylin.

### Pathologic assessment

FOXP3, CD8, HMGB1 and LC3B expressions were independently assessed by a trained histologist (PD). For CD8 and FOXP3 staining, slides were digitized with a slide scanner (Hamamazou, Massy, France) and processed with CaloPyx (Châtillon, France) software enabling whole slide quantification. For HMGB1 staining, the pattern of expression (nuclear or not) was evaluated in tumor cells: strong nuclear staining of at least 50% of the tumor cells was considered positive for HMGB1 tumor expression (see results and Supplemental Figure 2). Concerning LC3B, the intensity of LC3B *puncta* in tumor cells and the percentage of tumor cells with detectable LC3B *puncta* were assessed: the tumor was considered positive if LC3B *puncta* was detectable in more than 10% of malignant cells.

### Statistical analyses

All data manipulation, analyses and graphing were performed within the R environment [40]. Prognosis value of the SNPs on the esophageal cancer specific survival (as defined as the time elapsed from the date of diagnosis and death from cancer) was evaluated by Cox proportional hazards modeling. Genotype incidences in clinical descriptors were calculated with Firth's penalized-likelihood logistic regression [41]. Distributions of FOXP3 and CD8 were assessed by linear modeling or the Cuzick test as appropriate. Estimation of an adequate cut-off that optimally separates the cohort in 2 groups of poor and good prognosis was performed according to the method described in Hothorn and Lausen [42]. Additionally, Cox regression models built after discretization of the marker based on the population median and terciles are evaluated and presented with their corresponding Kaplan Meier survival curves. Estimated parameters are accompanied by their 95% confidence intervals and *p* values are two-tailed and considered significant when less than 0.05.

## SUPPLEMENTARY MATERIAL FIGURES


