# Supplementary material for: Negative prognostic impact of regulatory T cell infiltration in surgically resected esophageal cancer post-radiochemotherapy
Source: Oncotarget. 2015 Jun 10;6(25):20840–50. doi: 10.18632/oncotarget.4428 (PMC4673233; doi:10.18632/oncotarget.4428)
Supplement: Supplementary file 1 [file oncotarget-06-20840-s001.pdf]

Negative prognostic impact of regulatory T cell infiltration in surgically resected esophageal cancer post-radiochemotherapy

Supplementary Material

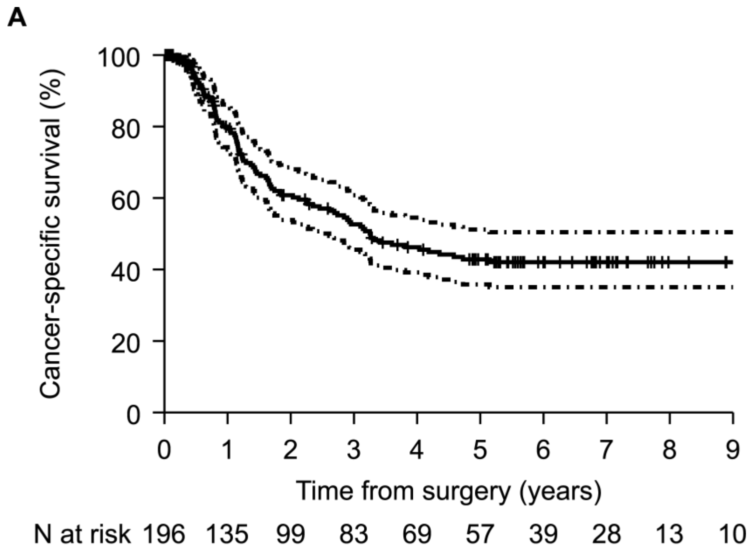

Supp Fig. 1: Cancer-specific survival in esophageal cancer. (A). Kaplan-Meier plot (alongside 95% confidential interval (CI) estimates) of the Cancer-specific survival estimated in the single-institution cohort of esophageal cancer patients (n = 196) treated with neo-adjuvant cisplatin-based radiochemotherapy. Median survival is estimated to be 3.26 years. Median follow-up of censored data is 5.3 years.

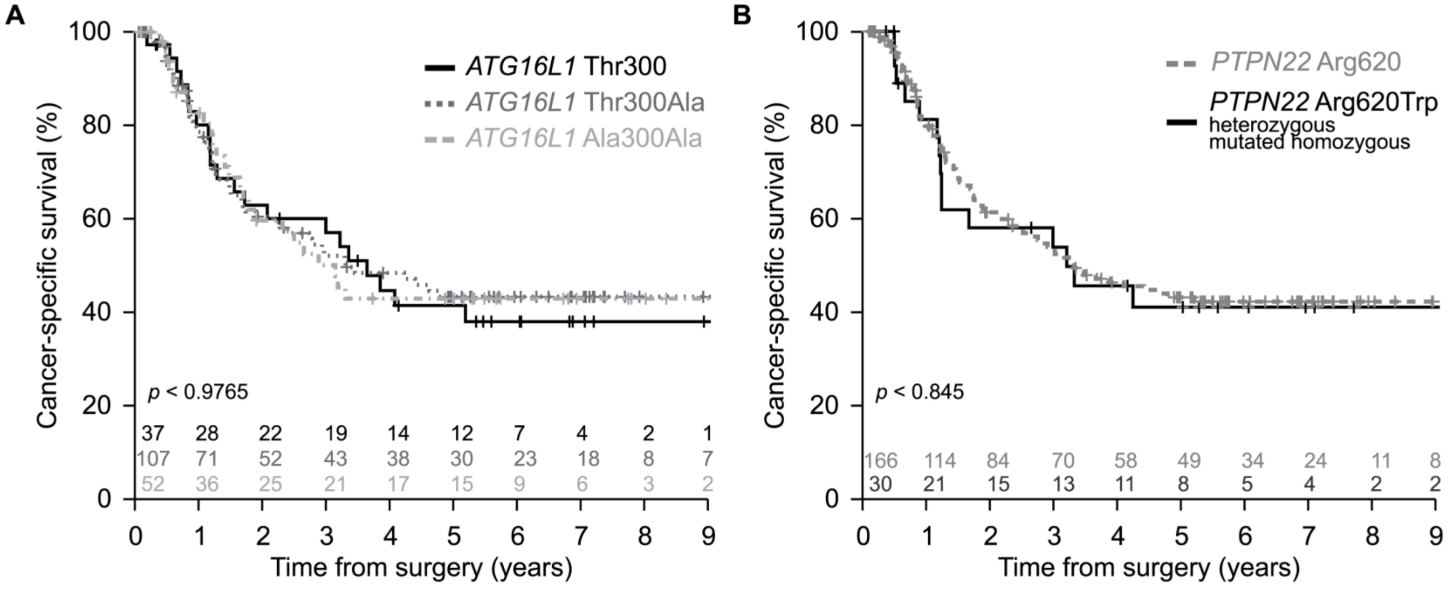

Supp Fig. 2: Impact of *ATG16L1* and *PTPN22* loss-of-function alleles on cancer-specific survival in esophageal cancer. (A) Cancer-specific survival Kaplan-Meier curves estimated in a cohort of esophageal cancer patients (n = 196) treated with neo-adjuvant cisplatin-based radiochemotherapy and bearing *ATG16L1* rs2241880 with AA (wild type, Thr300) or AG (heterozygous, Thr300Ala) or GG (mutated homozygous, Ala300Ala) genotype. **(B)** Cancer-specific survival Kaplan-Meier curves estimated in a cohort of esophageal cancer patients (n = 196) treated with neo-adjuvant cisplatin-based radiochemotherapy and bearing *PTPN22* rs2476601 with GG (wild type, Arg620) or AG (heterozygous, Arg620Trp) + AA (mutated homozygous, Trp620Trp) genotypes. Statistical significance was determined by likelihood ratio test (LRT).

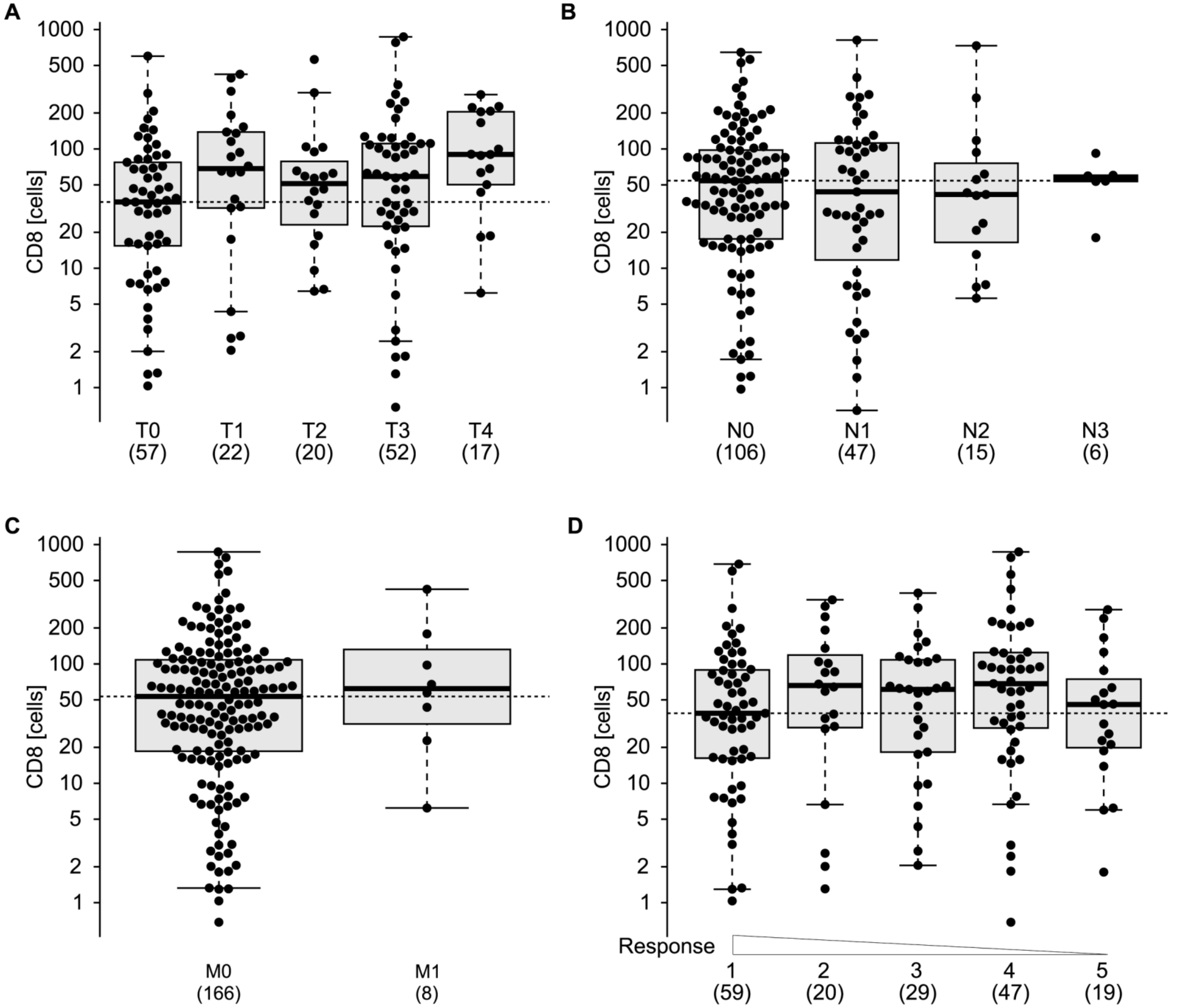

Supp Fig. 3: Distribution of CD8^{+} T regulatory infiltrating lymphocytes according to clinical parameters and treatment response. Enumeration of CD8^{+} cells according to tumor staging at the time of surgery **(A)**, lymph nodes involvement **(B)**, presence of metastasis **(C)**, tumor regression grading (TRG) **(D)**.

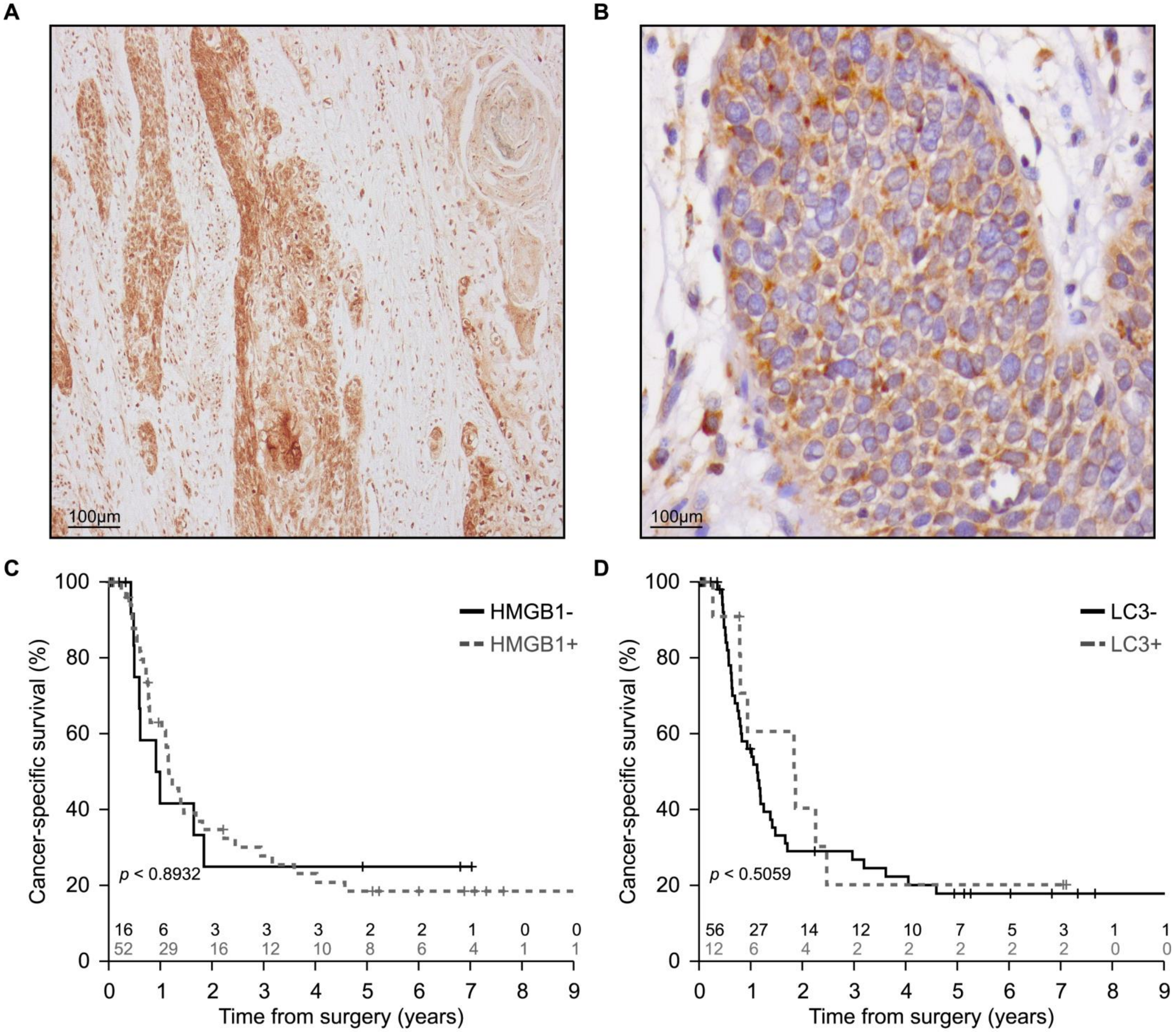

Supp Fig. 4: Immunohistochemical analysis of the level of expression of nuclear HMGB1 and cytoplasmic LC3 *puncta* in esophageal cancer patients. (A) Representative picture of immunohistochemical staining of primary paraffin embedded esophageal carcinoma using HMGB1 specific antibody. Positive cells are stained brown. **(B)** Representative picture of immunohistochemical staining of primary paraffin embedded esophageal carcinoma using LC3 specific antibody. Positive cells are stained brown. Magnification x30. **(C,D)** Cancer-specific survival Kaplan-Meier curves. Distribution according to the level of expression of nuclear HMGB1 **(C)** and cytoplasmic LC3 *puncta* **(D)** in esophageal cancer patients (n = 68).
